# Necrotizing Pneumonia Complicated by Bartonella-Associated Culture-Negative Endocarditis in an Immunocompetent Adult

**DOI:** 10.7759/cureus.93851

**Published:** 2025-10-05

**Authors:** Teja Koi, Patrick Yue, Ashley Huggett

**Affiliations:** 1 Department of Internal Medicine, William Carey University College of Osteopathic Medicine, Hattiesburg, USA; 2 Division of Infectious Diseases, Augusta University Medical College of Georgia, Augusta, USA

**Keywords:** bartonella endocarditis, blood culture-negative endocarditis, bronchopleural fistula, late latent syphilis, necrotizing pneumonia, pulmonary empyema

## Abstract

*Bartonella*-related culture-negative endocarditis (CNE) is an infrequent and difficult diagnosis, due to the organism’s unique growth characteristics and frequent negative cultures. We describe a case of a 42-year-old previously healthy male with necrotizing pneumonia complicated by empyema and bronchopleural fistula. Despite signs of systemic inflammation and infection, multiple blood cultures remained negative. Our diagnosis was made with serologic testing, which revealed elevated *Bartonella *antibody levels, and echocardiographic findings of endocarditis. *Bartonella *species are zoonotic, time-dependent, Gram-negative bacteria most often transmitted by arthropod vectors. Cats often serve as reservoirs of the organism. Because of ambiguous clinical manifestations, early recognition is challenging unless there is initial suspicion of a zoonotic etiology. We hope this case emphasizes thinking of *Bartonella *and other zoonotic pathogens in CNE when routine cultures do not provide a diagnosis. Serologic testing and imaging studies are invaluable in the diagnostic process. Rapid identification and the use of appropriate antimicrobial therapy are essential to avoid significant complications and improve patient care.

## Introduction

Infective endocarditis (IE) is a severe infection resulting from inflammation of the endocardial surface, most notably the cardiac valves. Blood culture-negative endocarditis (CNE) occurs in 5-31% of IE cases, frequently due to prior antibiotic use, difficult-to-culture organisms, or atypical zoonotic pathogens [1-3]. *Bartonella *spp. are Gram-negative, facultative intracellular bacteria increasingly recognized as causes of CNE, particularly in patients with pertinent epidemiologic exposures [4]. *Bartonella henselae *is primarily transmitted via domestic cats, while *B. quintana* is associated with human body lice [5].

Both species can cause subacute or chronic infection, often presenting with nonspecific systemic symptoms, such as malaise, fatigue, low-grade fevers, or weight loss, which can delay diagnosis. Complications may include valvular damage, heart failure, and systemic embolization. Because *Bartonella *spp. are intracellular and slow-growing, standard blood cultures often fail to detect infection, making serologic testing and echocardiography essential [5]. This case highlights an unusual presentation of *Bartonella*-associated CNE with concurrent necrotizing pneumonia and empyema in an adult male.

## Case presentation

A 42-year-old man with a history of asthma presented to the emergency department with worsening dyspnea for the past two weeks, a productive cough with yellow sputum, and intermittent fatigue. He denied fevers, weight loss, or chest pain. He has two pet indoor-outdoor cats, but he denied any recent travel, alcohol use, drug use, homelessness, or incarceration.

On arrival, the patient was tachypneic (RR: 26), tachycardic (HR: 112), and hypoxic (88% on room air, improving to 98% on a 2L nasal cannula). The physical exam revealed decreased breath sounds bilaterally; no murmurs on cardiac exam. Relevant laboratory findings included leukocytosis, microcytic anemia, thrombocytosis, hyponatremia, elevated alkaline phosphatase, and hypothyroidism (Table 1). The patient's initial labs suggested a very low hemoglobin; however, he did not require a transfusion as repeat labs indicated a hemoglobin level of >7 g/dL and the patient did not present with any signs of active bleeding. Serology for syphilis was reactive by rapid plasma reagin (RPR) (1:64). HIV and hepatitis panels were non-reactive.

**Table 1:** Initial laboratory findings MCV: mean corpuscular volume; RPR: rapid plasma reagin; TSH: thyroid-stimulating hormone

Test	Result	Reference Range
WBC	18.8 K/μL	4.0-11.0 K/μL
Hemoglobin	6.5 g/dL	13.5-17.5 g/dL
MCV	57.1 fL	80-100 fL
Platelets	586 K/μL	150-400 K/μL
Sodium	130 mmol/L	135-145 mmol/L
CO₂	18 mmol/L	22-28 mmol/L
TSH	29.231 mIU/L	0.4-4.5 mIU/L
Free T4	0.64 ng/dL	0.7-1.9 ng/dL
RPR HIV	1:64, Negative	Non-reactive, non-reactive

**Figure 1:**
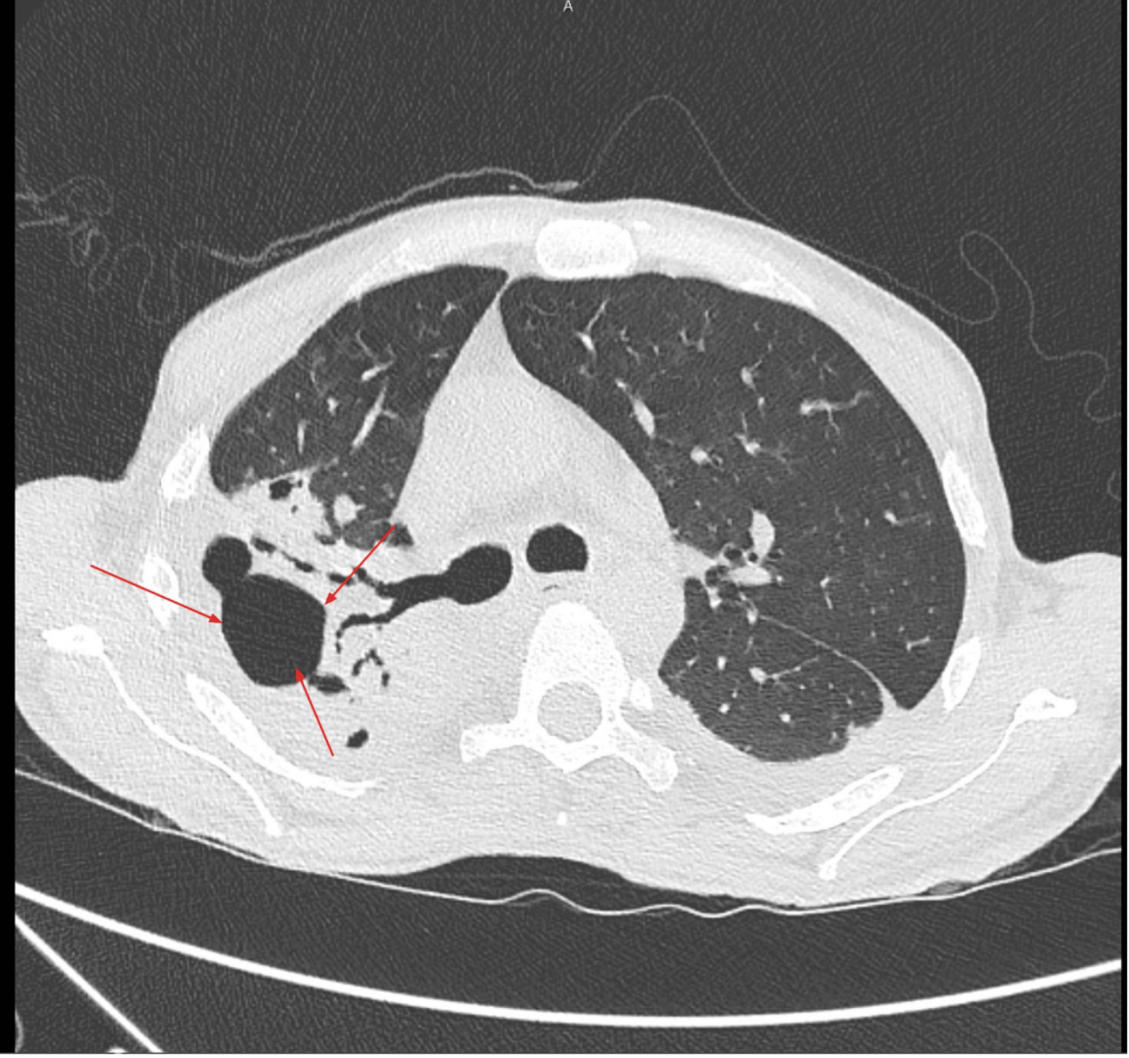
Axial chest CT shows a large, thick-walled cavitary lesion in the right upper lobe with multiple internal air-fluid levels and surrounding parenchymal consolidation

Contrast-enhanced CT chest/abdomen/pelvis imaging revealed bilateral multifocal consolidations with cavitation in the right upper lobe (6.0 x 3.2 x 5.1 cm) and large necrotic abscesses, which were consistent with necrotizing pneumonia (Figure 1). There was evidence of a right-sided bronchopleural fistula and a large loculated left pleural effusion (Table 2). The procedure to place a left-sided pigtail catheter for drainage was performed by pulmonology; analysis confirmed empyema with neutrophilic predominance, and cultures grew *Streptococcus constellatus*. Bronchoalveolar lavage grew methicillin-resistant *Staphylococcus aureus *(MRSA).

**Table 2:** Imaging findings (CT chest with contrast)

Location	Finding	Size
Right upper lobe	Necrotic abscess	6.0 x 3.2 x 5.1 cm
Right middle lobe	Bronchopleural fistula	Small
Left pleural space	Loculated empyema	Multiloculated

Wide-spectrum antibiotics, vancomycin, cefepime, and metronidazole, were given, yet inflammatory markers remained elevated. Blood cultures were persistently negative. A transesophageal echocardiogram (TEE) was ordered, and it showed a mobile echodensity on the aortic valve with trace aortic regurgitation and reduced EF (25-30%) (Table 3).

**Table 3:** Echocardiographic findings TEE: transesophageal echocardiogram; TTE: transthoracic echocardiogram

Modality	Findings
TTE	EF 51-55%, poor valve visualization
TEE	EF 25-30%, mobile mass on aortic valve, trace AR

Given the patient’s history of cat exposure and negative cultures, zoonotic testing was pursued. *Bartonella *serologies returned positive: *B. henselae* IgG 1:512 and *B. quintana *IgG 1:1024. Q fever serologies were negative (Table 4).

**Table 4:** Microbiology and serologies

Test	Result
Pleural fluid culture	Streptococcus constellatus
BAL culture	MRSA
Blood cultures	Negative x multiple
Bartonella serology	*B. henselae* IgG 1:512, *B. quintana* IgG 1:1024
Q fever serology	Negative

Based on the clinical and serologic data, the patient was diagnosed with *Bartonella*-associated CNE. The patient directed therapy with doxycycline 100 mg BID. Rifampin was avoided due to potential drug-drug interactions with alprazolam, which he was taking for his anxiety. Benzathine penicillin was changed to continuous infusion IV penicillin G due to coexisting latent syphilis. The patient was set to receive a six-week course of antibiotic therapy and follow-up at the outpatient clinic to reassess.

## Discussion

This case highlights the diagnostic challenges associated with CNE, particularly when *Bartonella *species are the cause. Although CNE represents a minority of IE cases, *Bartonella *has emerged as a leading etiology when epidemiologic exposures are present [2,3]. Its intracellular biology and atypical growth requirements explain persistently negative blood cultures, which can delay diagnosis and treatment.

*B. henselae *and *B. quintana *are small, intracellular, Gram-negative bacilli with differentiated epidemiologic features. *B. henselae *infection is classically known for being associated with exposure to domestic cats with close contact, such as scratches, bites, and contact with cat fleas, which are vectors of the bacteria. *B. quintana *is classically affiliated with poor hygiene, homelessness, and infestation with the human body louse, many of whom are socioeconomically disadvantaged [4,5]. Both species can cause subacute or chronic infection, including IE that can develop in a subacute manner over several weeks to months [4,5]. *Bartonella *IE is complicated to recognize early because the clinical symptoms often start insidiously with non-specific systemic symptoms of malaise, weight loss, and low-grade fevers.

Diagnosis requires integration of serology, imaging, and molecular methods where available. IgG titers ≥1:800 are strongly predictive of *Bartonella *endocarditis in the appropriate context [6-9]. Polymerase chain reaction (PCR) of blood or excised valve tissue can provide confirmatory evidence, but it is not always available, and sensitivity varies. Next-generation sequencing (NGS) of microbial 16S rDNA is an emerging tool in cases where cultures and serologies are inconclusive, especially in immunocompromised hosts [10]. In this patient, markedly elevated *Bartonella *titers along with TEE-confirmed vegetations supported the diagnosis.

Management requires prolonged antimicrobial therapy. Doxycycline for at least six weeks is standard, often combined with an aminoglycoside or rifampin for synergistic activity [11,12]. Rifampin was contraindicated here due to drug interactions, so doxycycline monotherapy was used. While doxycycline alone is generally considered less desirable, the treatment was administratively acceptable given that the patient was clinically stable and not exhibiting clinical complications related to embolization. The patient was hospitalized for a prolonged period due to necrotizing pneumonia and empyema, for which they required hospitalization, an ideal situation to ensure completing the full course of antibiotics as an inpatient.

Necrotizing pneumonia is an aggressive pulmonary process marked by cavitation, liquefactive necrosis, and parenchymal destruction. It is most commonly linked to highly invasive organisms such as *S. aureus*, *Klebsiella pneumoniae*, and members of the *Streptococcus anginosus *group and is typically encountered in patients who are critically ill or immunocompromised [13,14]. When described in the context of endocarditis, necrotizing pneumonia is usually secondary to septic emboli originating from infected valves rather than a concurrent destructive pulmonary infection. The presence of widespread parenchymal necrosis, empyema, and bronchopleural fistula alongside CNE, therefore, represents the intersection of two severe disease processes that rarely occur together.

What makes this case particularly distinct is the coexistence of *Bartonella*-associated CNE with necrotizing pneumonia in an otherwise healthy host. Although more than one hundred cases of *Bartonella *endocarditis have been published, most involve isolated cardiac disease or patients with well-recognized risk factors, such as homelessness, HIV infection, or other immunosuppressive states [5,7]. To date, *Bartonella *has not been reported in association with necrotizing pneumonia as a parallel process. The combination of destructive lung infection requiring invasive drainage and valve involvement with persistently negative blood cultures broadens the known clinical spectrum of *Bartonella *infection. It also highlights the importance of recognizing that overlapping pulmonary and cardiac manifestations may reflect two distinct but simultaneous infectious pathways, particularly when initial diagnostic testing does not point to a single unifying cause [5,13,14].

## Conclusions

*Bartonella *species are important causes of CNE and should remain a key diagnostic consideration in patients with negative blood cultures, unexplained valvular vegetations, and a history of animal exposure, particularly to cats. This case adds to the literature by documenting the rare coexistence of *Bartonella *endocarditis with necrotizing pneumonia, empyema, and a bronchopleural fistula in an adult male - an unusual presentation that broadens the recognized spectrum of *Bartonella *infection. To conclude, this case highlights the need for vigilance in diagnosing culture-negative endocarditis and awareness of the rare complications that may accompany it.
